# Healthcare costs in relation to kidney function among older people: the SCOPE study

**DOI:** 10.1007/s41999-024-01086-8

**Published:** 2024-11-13

**Authors:** Francesco Balducci, Mirko Di Rosa, Regina Roller-Wirnsberger, Gerhard Wirnsberger, Francesco Mattace-Raso, Lisanne Tap, Francesc Formiga, Rafael Moreno-González, Tomasz Kostka, Agnieszka Guligowska, Rada Artzi-Medvedik, Itshak Melzer, Christian Weingart, Cornel Sieber, Johan Ärnlöv, Axel C. Carlsson, Fabrizia Lattanzio, Andrea Corsonello, the Screening for CKD among Older People across Europe (SCOPE) study investigators, Francesco Balducci, Mirko Di Rosa, Fabrizia Lattanzio, Andrea Corsonello, Silvia Bustacchini, Silvia Bolognini, Paola D’Ascoli, Raffaella Moresi, Giuseppina Di Stefano, Cinzia Giammarchi, Anna Rita Bonfigli, Roberta Galeazzi, Federica Lenci, Stefano Della Bella, Enrico Bordoni, Mauro Provinciali, Robertina Giacconi, Cinzia Giuli, Demetrio Postacchini, Sabrina Garasto, Annalisa Cozza, Francesco Guarasci, Sonia D’Alia, Romano Firmani, Moreno Nacciariti, Paolo Fabbietti

**Affiliations:** 1 Centre for Biostatistics and Applied Geriatric Clinical Epidemiology, Italian National Research Center on Aging (IRCCS INRCA), Ancona and Cosenza, Ancona, Italy; 2 Department of Internal Medicine, Medical University of Graz, Graz, Austria; 3 Section of Geriatric Medicine, Department of Internal Medicine, Erasmus MC University Medical Center Rotterdam, Rotterdam, The Netherlands; 4 Geriatric Unit, Internal Medicine Department and Nephrology Department, Bellvitge University Hospital, IDIBELL, L’Hospitalet de Llobregat, Barcelona, Spain; 5 Department of Geriatrics, Healthy Ageing Research Centre, Medical University of Lodz, Lodz, Poland; 6 The Recanati School for Community Health Professions at the Faculty of Health Sciences at Ben-Gurion University of the Negev, Beersheba, Israel; 7 Maccabi Healthcare Services, Southern Region, Tel Aviv, Israel; 8 Department of General Internal Medicine and Geriatrics, Krankenhaus Barmherzige Brüder Regensburg and Institute for Biomedicine of Aging, Friedrich-Alexander-Universität Erlangen-Nürnberg, Erlangen, Germany; 9 School of Health and Welfare, Dalarna University, Falun, Sweden; 10 Division of Family Medicine and Primary Care, Department of Neurobiology, Care Sciences and Society (NVS), Karolinska Institutet, Stockholm, Sweden; 11 Department of Pharmacy, Health and Nutritional Sciences, University of Calabria, Cosenza, Italy

**Keywords:** Chronic kidney disease, Healthcare costs, Resources consumption, Older people, Multicentre

## Abstract

**Purpose:**

In this study, a comprehensive analysis of costs of chronic kidney disease (CKD) was performed, to understand factors associated with the economic burden of the disease in a multicentre international framework.

**Methods:**

The impact on costs of demographics, socio-economics, clinical, and functional variables was tested in 2204 subjects aged 75 years or more attending outpatient clinics in Europe using a multicentre 2-year prospective cohort study. By means of collected resources consumption and unit cost data a comprehensive cost database was built and then investigated using multilevel regression modeling.

**Results:**

Overall, hospitalization, medications and specialist visits were the main cost items, with a notable variability among countries. Estimated yearly costs were 4478€ ± 9804€, rising up to 6683€ ± 10,953€ for subjects with estimated Glomerular Filtration Rate (eGFR) < 30. Costs increased significantly according to the severity of the disease, gender and age. Clinical and functional covariates were also significantly associated with CKD-related total costs, even after correcting for the inter-country variability.

**Conclusion:**

Findings corroborate the importance of multidimensional assessment of participants with CKD, as multimorbidity and functional disability produce a detrimental impact on participant’s prognosis and cost of care. Preservation of functional impairment and adequate management of comorbidities may thus help decreasing the overall consumption on health care resources in CKD patients, especially in older people.

**Supplementary Information:**

The online version contains supplementary material available at 10.1007/s41999-024-01086-8.

## Key summary points

**Aim:**

To understand factors associated with the economic burden of chronic kidney disease.

**Findings:**

Costs increased significantly according to the severity of the disease, gender and age. Clinical and functional covariates were also significantly associated with CKD-related total costs, even after correcting for the inter-country variability.

**Message:**

Preservation of functional impairment and adequate management of comorbidities may thus help decreasing the overall consumption on health care resources in CKD patients, especially in older people.

**Supplementary Information:**

The online version contains supplementary material available at 10.1007/s41999-024-01086-8.

## Introduction

Chronic Kidney Disease (CKD) is a severe progressive disease projected to reach a global prevalence of 8–14% worldwide [1, 2], with steeper increases in the last decades due to population aging and increased prevalence of commonest risk factors, such as hypertension and type 2 diabetes mellitus [2]. Consequently, CKD-related economic burden on National Health Systems (NHS) is globally rising, mainly because of decreased overall health status and quality of life of patients with kidney function deterioration [2]. Among factors contributing to CKD-related costs, socioeconomic characteristics like male sex, low income or unemployment are also associated with increased CKD-related mortality [3]. Among clinical factors, degree of kidney function deterioration significantly impacts health-related quality of life, treatments and inherent costs [4]; indeed, CKD burden tends to increase with decreasing estimated Glomerular Filtration Rate (eGFR) and worsening kidney function [2, 5–8]. However, early diagnosis can be challenging, as participants with mild-moderate CKD are often asymptomatic and unaware of the risk of disease progression [9]; this leads to delayed diagnosis and progression to advanced CKD stages, when there are limited opportunities to avoid complications and improve quality of life and life expectancy [9]. Patients with kidney failure (CKD stage 5) are at exceptionally high risk of requiring kidney replacement therapies (KRT), such as dialysis and kidney transplantation, which increase both social and economic burden [10, 11]. Moreover, patients with advanced CKD are at higher risk of cardiovascular complications, with increased hospitalization rates and economic costs [11–13].

The above considerations are especially relevant for older populations, where CKD has per se a strong relationship with aging and age-related multimorbidity [5, 14, 15], which contribute to increased costs. CKD-related comorbidity and multimorbidity amplify the complexity of the disease, healthcare utilization, length of hospital stays, and impact prognosis [7, 16–19]. In this regard, recent studies have shown that direct costs of hospital admission of CKD patients with diabetes are considerably higher than those without diabetes [20, 21]; similarly, the per patient per year and inpatient costs of CKD increased in patients with comorbid diabetes mellitus, cardiovascular disease, or heart failure [8, 22]. The presence of physical disability may also complicate the CKD course and the patterns of CKD-related multimorbidity [14, 23], thus representing an additional cost multiplier in this context. A comprehensive approach to geriatric patients with CKD is then deemed necessary to tailor appropriate treatment strategies and decrease the burden on healthcare systems [24].

The economic burden of CKD is also dependent on the geographic setting where the patients are located, due to the different efficiency levels of NHSs—in turn related to Gross Domestic Product (GDP) [25, 26], differentiated costs of treatments, and heterogeneity of healthcare delivery [2, 26, 27]. Indeed, Germany, Sweden, and Spain had the highest median annual costs, while Poland was characterized by the lowest median costs [2]; in Italy, the median costs ranged from 1553€ in stage G3a to 4,632€ in stage G5, in the absence of KRTs. The annual cost was further increased in patients developing complications associated with CKD, such as stroke, acute kidney injury, haemodialysis or kidney transplant, and cardiovascular disease [2].

In spite of the variability of dimensions involved, most previous studies on CKD costs were performed in patients with advanced disease only or did not consider the multidimensional components of CKD burden, including multimorbidity and functional disability.

In light of the above, the objective of this study was to perform a Cost Of Illness (COI) analysis for CKD among older people attending outpatient clinics in Europe, in an attempt to understand factors associated with the economic cost of CKD in a multicentre international framework.

## Methods

### Study population

Data were collected in the context of the Screening for CKD among Older People across Europe (SCOPE) study (European Grant Agreement no. 436849), a multicentre 2-year prospective cohort study involving people older than 75 years attending outpatient services in participating institutions in seven countries (Austria, Germany, Israel, Italy, the Netherlands, Poland and Spain). The study protocol was approved by ethics committees at all participating institutions and complies with the Declaration of Helsinki and Good Clinical Practice Guidelines. After signing a written informed consent, study participants underwent a comprehensive geriatric assessment (CGA), including demographic data and socioeconomic status, physical examination, medical history, use of medications, healthcare resource consumption, caregiver burden, and blood and urine sampling.

Exclusion criteria were: end-stage renal disease or dialysis at time of enrollment; history of solid organ or bone-marrow transplantation; active malignancy within 24 months prior to screening or metastatic cancer; life expectancy less than 6 months (based on the judgment of the study physician after careful medical history collection and diagnoses emerging from examination of clinical documentation exhibited); severe cognitive impairment (Mini Mental State Examination (MMSE) < 10); any medical or other reason (e.g., known or suspected patients’ inability to comply with the protocol procedure) in the judgment of the investigators, that the patient was unsuitable for the study; unwilling to provide consent and limited possibility to attend follow-up visits [28]. Methods of the SCOPE study have been extensively described elsewhere [28].

**Fig. 1:**
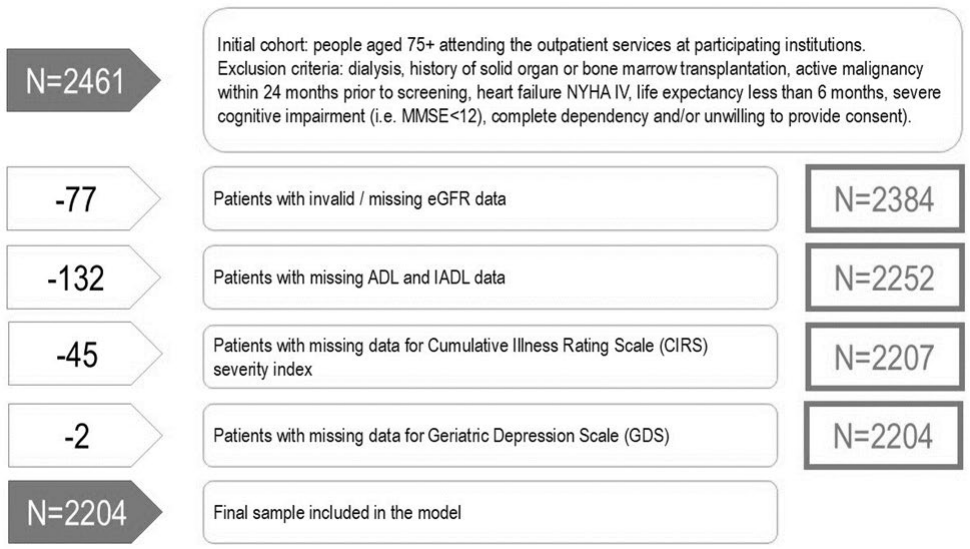
Flow-chart of data

Overall, 2,461 participants were enrolled in the study from August 2016 to March 2018. Of them, 2384 participants had available eGFR data. After excluding participants with missing data in other clinical study variables, the final sample included 2204 participants with complete data. There was no significant difference in the demographic characteristics of the participants with missing data, with respect to the final sample included in the study, a part from caregiving needs, which were higher for the 256 excluded participants (Fig. 1, and Table S7 in supplementary material).

### Study variables

Study variables included the demographic characteristics of participants, such as sex, age, marital status (single, married or cohabiting, divorced, widowed). Living conditions (alone or with others) and smoking habit (being a current or a former smoker) were also included in the analysis.

The socio-economic profiles were considered by taking years of education into account. The occupational level participants had in the last work they did was recoded into broad skill levels (Low: elementary occupations; Medium: clerical, services, skilled workers and operators, craft workers; High: managers, professionals and technicians; and Other) on the basis of International standard classification of occupations (ISCO) [29].

Participants were also directly asked to state their economic status (recoded in “good or very good”, “sufficient”, “mediocre or bad”) and if their income was enough to cover for their living expenditures (such as rent, shopping, electricity, drugs, and so forth).

Overall comorbidity was measured by Cumulative Illness Rating Scale (CIRS-G) severity index [30]. Selected comorbidities usually associated with CKD (diabetes, hypertension, atrial fibrillation anemia and hyperparathyroidism) were separately considered in the analysis. The number of prescribed medications was also calculated and an analytical variable to identify polypharmacy was coded as taking five or more medications and included in the analysis. A CGA was performed and included the MMSE, available and validated in all languages of participating countries, for cognitive status [31], the 15-items Geriatric Depression Scale – Short Form (GDS-SF) for mood (participants scoring 5 or more were considered affected by clinically meaningful depressive symptoms) [32], available and validated in all languages of participating countries, Basic Activities of Daily Living (BADL) (bathing and showering, personal hygiene and grooming, dressing, toilet hygiene, functional mobility, self-feeding) [33] and Instrumental Activities of Daily Living (IADL) for physical performance (meal preparation, household chores, medication management, shopping and transportation, managing communication) [34]. BADL and IADL ranges from 0 (independence) to 4 (complete dependence). The need and hours of caregiving were also recorded.

Assessment of kidney function was based on measurement of serum creatinine levels and urinary albumin-to-creatinine ratio (ACR); serum creatinine was measured at local level using standard methods. Creatinine-based eGFR was calculated using the Berlin Initiative Study 1 (BIS1) equation [35]. Other specifications and classifications were tested (see supplementary tables S8-S13 for a comparison using four levels eGFR [36, 37]and with the EKFC formula [38]). Urinary ACR was measured by urine spot analysis and expressed in mg/g. Both eGFR and ACR were categorized according to “Kidney Disease: Improving Global Outcomes” (KDIGO) guidelines [39].

### Resource consumption and cost data

In addition to collecting clinical data of participants, the consumption of healthcare resources was recorded to identify and evaluate the costs components. An ad-hoc Resource Use Questionnaire was used to collect information referring to a 6-month recall time-frame [28]. Data were detailed on previous physician visits (general practitioners-GPs-, specialists), emergency department visits, use of diagnostic tests and specialist clinic procedures, use of care services (e.g., nurse home visit, physiotherapy, home help, social transport, day care center), hospital admissions (number and duration of hospitalization). Detailed information was also collected on laboratory tests performed (hematology, biochemistry, urinalysis) and medications taken by the participants. Both current and prescribed drugs were recorded with their name and formulation, route, unit dose, number of units per day and days per week (Supplementary Material—Tables S1 and S2).

To aggregate the items in natural units (e.g., number of visits or hospital days) into an overall healthcare cost it was necessary to multiply the resources consumed for their unit prices (i.e., unit cost of a specific drug, cost a GP visit etc.). Each cost item pertained to broader categories of costs, which in turn were classified into direct medical costs or non-medical costs. Direct medical costs included: GP’s visits, specialist visits, use of emergency department, hospitalization, drugs, instrumental and laboratory (hematology, biochemistry and urine analysis) diagnostic tests. Direct non-medical costs included: nurse home visits, physiotherapy, home help, social transport and day care center. Cost of professional or informal caregiving were also considered. Indirect costs (productivity loss) and other societal costs were not considered in the analysis. For each information above participants and/or caregivers were asked to state if the resource was explicitly a CKD-related one and also to indicate the type —if any—of reimbursement received for the cost bore (Supplementary Material—Table S2).

The total number of days of hospitalization has been obtained comparing discharge and admission dates. In regards to diagnostic tests, the ten most frequently used ones were selected by International Classification of Diseases, 9th Revision, Clinical Modification (ICD-9 CM) codes (Electrocardiogram, Routine X-ray of chest NOS, Echocardiography, Diagnostic ultrasound of full abdomen, Computerized axial tomography of head, Skeletal X-ray of pelvis and hip, Carotid Color Doppler ultrasonography, Skeletal x-ray of thigh, knee, and lower leg, Urine culture, Color Doppler ultrasonography of kidneys and adrenal glands). Similarly, the ten most frequently prescribed drugs were selected using ATC codes (ACE inhibitors, plain (C09AA), Angiotensin II antagonists, plain (C09CA), Beta blocking agents, selective (C07AB), Dihydropyridine derivatives (C08CA), HMG CoA reductase inhibitors (C10AA), Platelet aggregation inhibitors excl. Heparin (B01AC), Proton pump inhibitors (A02BC), Sulfonamides, plain (C03CA), Thyroid hormones (H03AA), Vitamin D and analogues (A11CC)). Erythropoiesis stimulating agents (ESAs) (ATC = B03XA01, B03XA02, B03XA03) were also considered in the analysis even if they were not among most frequently prescribed medications. Finally, for each diagnostic test/drug taken, the total unit doses per week, and per 6 months (avg. 26 weeks) have been computed merging and looping data from diagnostic test/drug level to participant level (supplementary material—Tables S3 and S4).

Unit cost data were provided in local currency by the medical centers for each category or resources. To take into account of variability of cost estimates, and to allow for the possibility of sensitivity analyses, partners were asked to provide a range of values (min–max per cost item) whenever possible. Individual source data were collected and recorded, conducting on-going quality checks. Source data were considered of high quality if: a) all the information was complete, b) data sources and value ranges provided, c) local currency and euro conversion (where applicable), d) no imputations necessary to convert costs into the requested unit. In spite of quality checks, computations were sometimes necessary to transform cost provided into desired units (e.g. converting hourly costs into daily costs, etc.).

Even if there were differences among participating countries, the direct medical cost components were obtained using the corresponding outpatient tariffs for i.e., laboratory tests, visits, diagnostic tests and so forth. Analogosly, hospitalization and Emergency Department (ED) tariffs were collected by the accounting departments of each medical center involved in the study. The costs for transport were collect on a per-km basis and subsequently harmonized by rescaling to a standard 10 km-round trip from home to the healthcare facility to perform visits or exams. For participants needing caregivers, the number of caregiving hours were multiplied by the hourly wage of a professional caregiver or, for informal caregivers, referring to national estimates found in the literature.

After revision, the final unit costs were converted into 2019 Euro, which is the last year with full data available for inflationusing the Harmonized Consumer Price Index [40].

### Analytic approach

First, a data cleaning and harmonization process was conducted. “Cost of CKD” was considered the primary outcome of the study. The cost variables and independent variables were checked for outliers and their distribution inspected. Variables were reclassified, recoded or standardized whenever necessary, and described using means and standard deviations (medians and Inter Quartile Ranges (IQR) for non-normally distributed variables).

As the cost of CKD is highly dependent on the severity of the disease, the differences in the outcome across the three eGFR groups (≥ 0, 30–59, and < 30 ml/min/1.73 m^2^) were investigated according to the exposure variables using Pearson’s Chi-squared test for categorical variables tests and ANOVAs for continuous variables. In case of CIRS-G, MMSE, BADL, IADL, where the distribution is not normal, dedicated Kruskal–Wallis (non-parametric) ANOVAs had been used. *p* values were reported: two-sided *p* values < 0.05 were deemed statistically significant.

After bivariate testing, multivariate cross-sectional regression models were built, to identify factors correlated with total cost of CKD disease. The selection of regressors followed a mixed method, aimed at including clinical variables which were relevant from a theoretical/explanatory point of view and at the same time excluding regressors which were highly correlated one another but un-correlated with the outcome at bivariate level, non-normally distributed or redundant.

After having tested ordinary least squares models for model selection, a Generalized Linear Model (GLM) framework with Gamma function and logarithmic link had been chosen as the best way to model the specific cost distribution.

Moreover, since the geographic effect of setting is crucial in this study, the regression models were improved by a multilevel (two-levels) regression model to account for the variability among various medical districts located in different countries. The hierarchy structure considered participants as nested in their countries [41]. Common indicators of goodness-of-fit (pseudo-R squared, Adjusted R-squared, Akaike Information Criterion-AIC and Bayesian information criterion-BIC) and other standard post-estimation procedures (inspection of residual, Variance Inflation Factors-VIF) were calculated. The statistical significance for this study was set at* p* < 0.05. Statistical analyses were conducted using the Stata/MP 18.0 Software Package for Windows (StataCorp., College Station, TX, USA).

## Results

### Characteristics of the sample and CKD levels

Overall, 55.8% of the 2204 participants included in the study were females. Mean age was 80 years, even if the largest part of participants were in the 75–79 class (55%, median age 79 years). 2.7% was ninety or more. Most participants were married or widowed and cohabiting (55%). Despite only 20% of participants had a university diploma, most of them had a medium or high occupational level and more than 90% of the sample declared to have at least a sufficient income level. More than 77% of the participants had comorbidities such as hypertension, 33.8% had hyperparathyroidism and 22% anemia. More than 67% of them used five or more concomitant medications (polypharmacy), and it was worth noting that only 32 out of 1463 (2.2%) patients with eGFR < 60 were prescribed ESAs. Nearly 60% of participant were in the middle class for eGFR (30–59), 33.6% had an eGFR of 60 or more and 7.3% (162 participants) had an eGFR < 30. Among medical centres, there were also differences among the mean eGFR scores, i.e., being higher in Israel and Poland and lower in Austria (Fig. S2 in supplementary material).

Stratifying by severity of CKD (i.e., eGFR levels), there were significant differences at bivariate level among all the variables considered, with the exception of income, GDS-SF, BADL, and caregiving hours (Table 1).

**Table 1:** Characteristics of participants based on CGA, by severity of CKD (eGFR levels)

	Overall (N = 2204)	eGFR ≥ 60 (N = 741)	eGFR: 30–59 (N = 1301)	eGFR < 30 (N = 162)	p
Female sex, *n* (%)	1229(55.8%)	464(62.6%)	700(53.8%)	65(40.1%)	< 0.001
Age, *n* (%)					< 0.001
75–79	1221(55.4%)	502(67.8%)	649(49.9%)	70(43.2%)	
80–84	678(30.8%)	195(26.3%)	430(33.1%)	53(32.7%)	
85–89	244(11.1%)	42(5.7%)	173(13.3%)	29(17.9%)	
90 +	61(2.78%)	2(0.3%)	49(3.8%)	10(6.2%)	
Marital status, *n* (%)					0.028
Single	120(5.4%)	47(6.3%)	58(4.5%)	15(9.3%)	
Married/cohab	1225(55.6%)	414(55.9%)	728(56.0%)	83(51.2%)	
Divorced	117(5.3%)	46(6.2%)	59(4.5%)	12(7.4%)	
Widowed	742(33.7%)	234(31.6%)	456(35.1%)	52(32.1%)	
Living alone, *n* (%)	541(24.6%)	168(22.7%)	318(24.4%)	55(34.0%)	0.010
Education (years), mean ± sd	11.26 ± 4.92	11.76 ± 5.16	11.13 ± 4.86	9.96 ± 3.85	
University diploma, *n* (%)	457(20.7%)	202(27.3%)	243(18.7%)	12(7.41%)	< 0.001
Occupational level, *n* (%)					< 0.001
Low	195(8.9%)	66(8.9%)	114(8.8%)	15(9.3%)	
Medium	871(39.5%)	254(34.3%)	529(40.7%)	88(54.3%)	
High	784(35.6%)	300(40.5%)	444(34.1%)	40(24.7%)	
Economic status, *n* (%)					< 0.001
Bad/mediocre	233(10.6%)	102(13.8%)	125(9.6%)	6(3.7%)	
Sufficient	829(37.6%)	307(41.4%)	485(37.3%)	37(22.8%)	
Good/very good	1142(51.8%)	332(44.8%)	691(53.1%)	119(73.5%)	
Not enough income, *n* (%)	207(9.4%)	75(10.1%)	119(9.2%)	13(8.0%)	0.634
eGFR (BIS1), mean ± sd	53.32 ± 14.66	68.59 ± 7.16	48.30 ± 8.13	23.83 ± 4.30	< 0.001
Proteinuria, *n* (%)					< 0.001
< 30	1603(72.7%)	635(85.7%)	937(72.0%)	31(19.1%)	
30–300	450(20.4%)	99(13.4%)	279(21.5%)	72(44.4%)	
> 300	151(6.9%)	7(0.9%)	85(6.5%)	59(36.4%)	
Smoke, *n* (%)					0.002
No	1269(57.6%)	453(61.1%)	734(56.4%)	82(50.6%)	
Former smoker	837(38.0%)	245(33.1%)	517(39.7%)	75(46.3%)	
Current smoker	98(4.4%)	43(5.8%)	50(3.8%)	5(3.1%)	
GDS-SF > 5, *n* (%)	312(14.2%)	101(13.6%)	188(14.5%)	23(14.2%)	0.877
Diabetes, *n* (%)	556(25.2%)	142(19.2%)	354(27.2%)	60(37.0%)	< 0.001
Hypertension, *n* (%)	1704(77.3%)	494(66.7%)	1056(81.2%)	154(95.1%)	< 0.001
Atrial fibrillation, *n* (%)	342(15.5%)	68(9.2%)	234(18.0%)	40(24.7%)	< 0.001
Anemia, *n* (%)	470(21.3%)	84(11.3%)	293(22.5%)	93(57.4%)	< 0.001
Hyperparathyr.,, *n* (%)	746(33.8%)	204(27.5%)	444(34.1%)	98(60.5%)	< 0.001
CIRS-G severity index, median (iqr)	1.5(0.6)	1.4(0.67)	1.5(0.61)	1.78(0.5)	0.001
MMSE, median (iqr)	29(3)	29(3)	29(3)	29(3)	0.045
BADL, median (iqr)	0(0)	0(0)	0(0)	0(2)	0.2603
IADL, median (iqr)	2(9)	0(5)	4(10)	11.5(14)	0.001
Polypharmacy (current), *n* (%)	1487(67.5%)	411(55.5%)	934(71.8%)	142(87.7%)	< 0.001
Polypharmacy (prescribed), *n* (%)	1085(49.2%)	257(34.7%)	701(53.9%)	127(78.4%)	< 0.001
Need of caregiving, *n* (%)	411(18.7%)	112(15.1%)	248(19.1%)	51(31.5%)	< 0.001
Hours of caregiving, mean ± sd	2.52 ± 12.81	2.31 ± 14.19	2.62 ± 12.63	2.57 ± 5.63	0.870

*eGFR* estimated Glomerular Filtration Rate, *CIRS-G* Cumulative Illness Rating Scale, *MMSE* Mini Mental State Examination (*MMSE*), *GDS-SF* Geriatric Depression Scale-Short Form, *BADL* Basic Activities of Daily Living, Instrumental Activities of Daily Living (IADL)

*p* values obtained using Pearson’s Chi-squared test for categorical variables tests and ANOVAs and Kruskal–Wallis ANOVAs for continuous variables

Two-sided *p* values < 0.05 were deemed statistically significant. Polypharmacy is defined as nr. of medications ≥ 5

### Analysis of CKD total costs and cost components

Overall, the mean total cost was 2239€ ± 4,902€ (4478€ ± 9804€ estimated yearly total cost) (Table 2). The main components of the total cost were hospitalization costs, medications and physician visits (GP plus specialists), which accounted for nearly 40%, 23%, and 18% of the mean total cost, respectively. The percent shares of laboratory tests, social transport, day care center and caregiving were comparatively low ( Table 2).

**Table 2:** Mean total cost across cost sub-components (6 months total, 2019 €)

Cost component	Mean	% on total cost	SD	Max
GP	131.5	5.9	158.1	2236.5
Specialist	280.5	12.5	461.3	10,038.6
ED	26.7	1.2	94.6	1750.0
Hospitalization	891.1	39.8	4149.6	61,099.3
Medications	514.9	23.0	869.5	18,723.8
Lab: haematology	3.2	0.1	7.2	97.6
Lab: biochemistry	20.0	0.9	42.3	498.1
Lab: urinanalysis	1.5	0.1	4.2	64.9
Nurse	160.0	7.1	1367.3	21,578.8
Physiotherapy	77.1	3.4	253.0	4100.0
Home help	52.8	2.4	200.0	4438.1
Social transport	2.8	0.1	25.7	780.0
Day care center	21.3	1.0	382.8	12,064.0
Proffessional caregiver	19.1	0.9	157.4	5250.0
Informal caregiver	7.7	0.3	39.3	563.2
Total cost	2,239	100.0	4902.2	64,185.1

*ED* Emergency Department, *GP* General Practitioner

The impact of the severity of CKD was relevant, with a mean total cost which rose to more than 3340€ for eGFR < 30 participants. Differences in mean are statistically significant with the exception of lab tests and caregiving sub-components (Table 3). As predictable, the mean total cost increased notably with age (from 1772€ for under-80s to 4358€ for over-90s), and it was slightly higher for women (+ 479€ with respect to males) (Table S5 in Supplementary Material).

**Table 3:** Mean total cost by eGFR levels (main categories, 6 months total, 2019 €)

	eGFR ≥ 60 (*N* = 741)	eGFR: 30–59 (*N* = 1301)	eGFR < 30 (*N* = 162)	*p*
Physician visits, mean ± sd	344.29 ± 365.18	436.67 ± 519.28	524.18 ± 479.42	< 0.001
Hospitalization, mean ± sd	702.03 ± 3373.78	983.95 ± 4516.16	1373.36 ± 4448.18	0.050
Medications, mean ± sd	395.85 ± 717.36	541.34 ± 936.62	846.92 ± 845.81	< 0.001
Lab tests, mean ± sd	22.58 ± 44.21	25.43 ± 48.93	28.96 ± 54.69	0.086
Care services, mean ± sd	151.07 ± 719.64	382.54 ± 1842.20	509.45 ± 2633.86	< 0.001
Caregiving, mean ± sd	28.10 ± 231.06	26.24 ± 114.51	25.07 ± 68.38	0.989
Diagnostic tests, mean ± sd	23.89 ± 54.80	30.52 ± 63.75	33.80 ± 82.71	0.020
Total cost, mean ± sd	1667.81 ± 3743.14	2426.69 ± 5353.51	3341.72 ± 5476.94	< 0.001

**Fig. 2:**
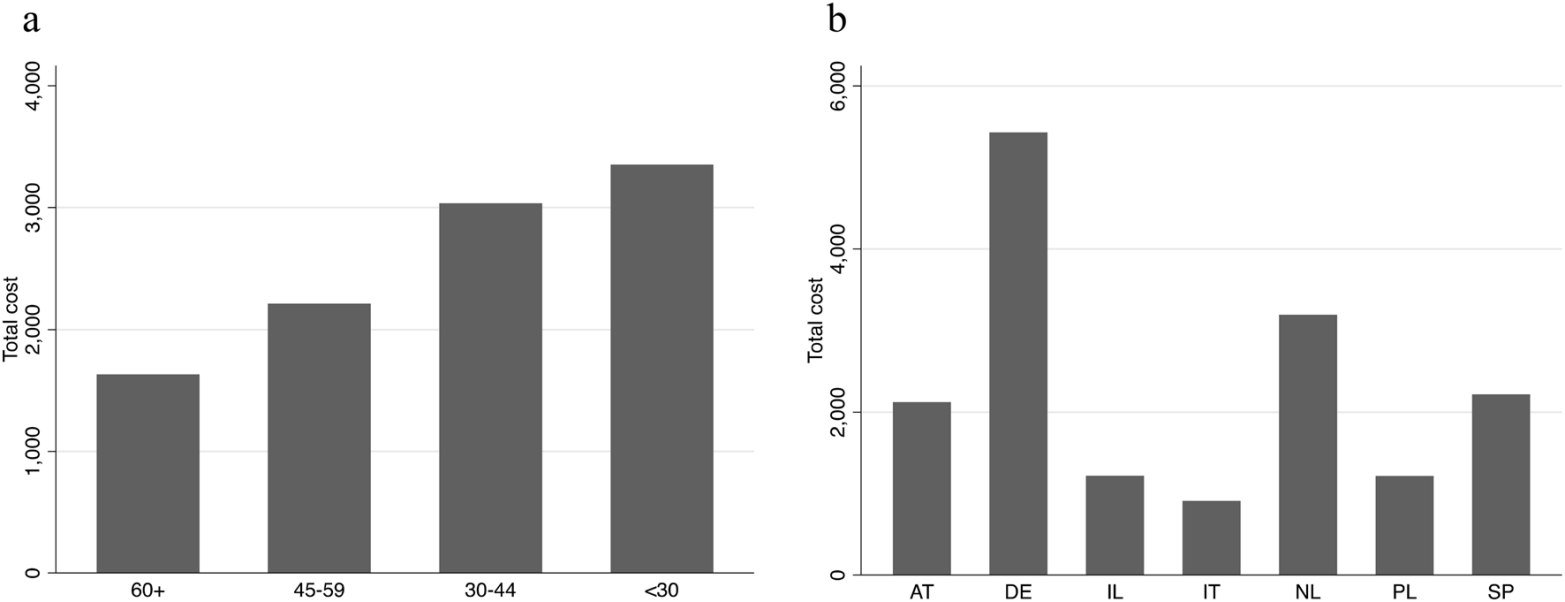
Mean total costs by eGFR level (**a**) and by medical center (**b**) (6 months totals, 2019 €). *eGFR* estimated Glomerular Filtration Rate

The mean total cost was higher in the medical centers located in Germany and The Netherlands. Mean costs where similar in Austria and Spain and lower in Italy, Poland and Israel (Fig. 2). When looking at cost sub-components, the greatest variability was due to hospitalization costs (i.e., a high unit price was recorded for a day in hospital in Germany). Germany is the country where the variability of costs was generally higher (Supplementary Material—Table S3 and Fig. S1).

### Factors associated with total costs of CKD

Considering the evidence of the previous paragraph, the association among socio-demographic and clinical factors and CKD total costs has been investigated with statistical analyses. At bivariate level, when looking at the matrix of pairwise correlations, the total cost was mildly and positively associated with female sex, age and marital status (widowed or divorced), eGFR and proteinuria levels, clinical conditions (diabetes, hypertension, atrial fibrillation), use of medications and home care. Instead, the correlation was negative with higher educational levels and MMSE scores (Supplementary Material—Table S6).

When costs were modeled in GLM regressions using eGFR as the only dependent variable, the association between the severity of disease and total costs was significant (“restricted” model: Table 4, model 1). By adding socio-economic regressors to the model the impact of eGFR slightly diminished, yet it was still significant. Other significant predictors were older age, fewer education years, not being married and living with others (model 2). Instead, when adding clinical and functional variables to the model, the measurement of eGFR lost its significance, which was captured by clinical correlates such as diabetes, hypertension, atrial fibrillation, CIRS-G severity index and IADL (model 3).

**Table 4:** Predictors of total cost: GLM-regression models

	GLM models (log link, gamma family)							
	(1)		(2)		(3)		(4)	
	Coef	SE	Coef	SE	Coef	SE	Coef	SE
eGFR (ref ≥ 60)								
30–59	0.375***	0.101	0.320***	0.095	0.098	0.101	0.022	0.104
< 30	0.695***	0.189	0.689***	0.180	0.109	0.207	– 0.024	0.220
Sex: female			0.117	0.096	0.331**	0.107	0.152	0.107
Age			0.042***	0.011	0.025*	0.012	0.007	0.013
Marital status (ref = single)								
Married			– 0.506*	0.209	– 0.446*	0.219	– 0.160	0.232
Divorced			– 0.056	0.261	0.067	0.271	– 0.035	0.280
Widowed			– 0.173	0.201	– 0.122	0.208	– 0.136	0.217
Living alone			– 0.368**	0.116	– 0.361**	0.122	0.032	0.138
Education (years)			– 0.047***	0.010	– 0.024*	0.011	– 0.022	0.012
Occupational level			0.087	0.048	0.075	0.051	0.014	0.052
Economic status			– 0.093	0.067	– 0.125	0.069	– 0.091	0.075
Proteinuria					0.131	0.088	0.079	0.091
Smoke					– 0.047	0.087	– 0.076	0.087
GDS-SF					– 0.119	0.132	0.075	0.136
Diabetes					0.283**	0.108	0.256*	0.110
Hypertension					0.296**	0.108	0.332**	0.111
Atrial fibrillation					0.340**	0.125	0.238	0.127
Anemia					0.234*	0.117	0.181	0.119
Hyperparathyroidism					– 0.198*	0.097	– 0.009	0.108
CIRS-G severity index					0.456***	0.114	0.091	0.128
MMSE					– 0.000	0.018	– 0.026	0.020
BADL					0.035	0.031	0.035	0.030
IADL					0.033***	0.009	0.030**	0.009
Medical center (ref = AT)								
DE							0.923***	0.219
IL							– 0.291	0.216
IT							– 0.947***	0.196
NL							0.394	0.213
PL							– 0.096	0.227
SP							0.001	0.210
Constant	7.419***	0.080	4.981***	0.896	4.525***	1.137	7353***	1.209
Observations	2204		2204		2204		2204	

*eGFR* estimated Glomerular Filtration Rate, *CIRS-G* Cumulative Illness Rating Scale, *MMSE* Mini Mental State Examination, *GDS-SF* Geriatric Depression Scale-Short Form, *BADL* Basic Activities of Daily Living, *IADL* Instrumental Activities of Daily Living, *GLM* Generalized Linear Model

*p < 0.05, **p < 0.01, ***p < 0.001

The results of the full model including country dummies showed that total costs were largely dependent on the countries where the medical centers were set. With respect to Austria (reference country), costs were comparatively higher in Germany and lower in Italy. Other countries’ coefficients were not significant, together with socioeconomic regressors (model 4). The only clinical covariates remaining significant were diabetes and hypertension. According to AIC and BIC criterion results (not reported), the full model has a slightly better explanatory power.

To fix the above estimates for country bias, a multilevel framework with participants nested in medical centers was deemed appropriate. Age, being female, diabetes, hypertension, atrial fibrillation, anemia, CIRS-G severity index, BADL, and IADLs were all positively associated with total CKD costs in the final model (total cost model: Table 5), which incorporated the geographic component within the modelling framework. When separating the regression models among cost subcomponents, some notable different behaviors appeared. The eGFR score became significant for classes higher than 30 for medications and 30–59 for physician visits, while it was not significant elsewhere. Not all the clinical variables which were significant for total costs had a significant impact for cost sub-items: e.g., diabetes, BADL or IADL for physician visits. For hospitalization there was a significant impact of GDS-SF and IADL. Diabetes and hypertension had still, however, a significant impact when medication costs were concerned.

**Table 5:** Predictors of total cost and costs items: multilevel models

	(1)		(2)		(3)		(4)		(5)	
	Total cost		Physician visits		Hospitalization		Medication		Cost of care	
	Coef	SE	Coef	SE	Coef	SE	Coef	SE	Coef	SE
eGFR (ref ≥ 60)										
30–59	0.009	0.054	0.087*	0.040	– 0.125	0.167	0.096	0.054	– 0.105	0.125
< 30	– 0.008	0.114	0.125	0.085	– 0.555	0.327	0.285**	0.110	– 0.071	0.261
Age	0.014*	0.006	– 0.009*	0.005	0.020	0.018	0.010	0.006	0.022	0.013
Sex: female	0.192***	0.058	0.004	0.043	– 0.141	0.177	0.261***	0.058	– 0.002	0.133
Marital status (ref = single)										
Married	– 0.010	0.112	– 0.006	0.084	0.190	0.317	– 0.006	0.114	– 0.079	0.234
Divorced	0.206	0.141	0.189	0.105	0.271	0.400	– 0.071	0.141	0.075	0.278
Widowed	0.050	0.108	0.064	0.081	– 0.034	0.297	– 0.090	0.110	– 0.009	0.215
Living alone	0.004	0.068	– 0.101*	0.051	0.059	0.214	0.028	0.067	0.412*	0.164
Education (years)	– 0.009	0.006	– 0.007	0.004	– 0.017	0.017	– 0.010	0.006	0.004	0.014
Occupational level	0.016	0.026	0.044*	0.019	– 0.096	0.085	0.033	0.026	– 0.031	0.063
Economic status	– 0.070	0.038	– 0.054	0.029	0.097	0.114	– 0.085*	0.039	0.107	0.083
Proteinuria	0.014	0.046	0.016	0.034	0.159	0.141	0.058	0.044	0.016	0.101
Smoking	– 0.010	0.043	– 0.035	0.032	– 0.212	0.137	0.069	0.043	– 0.062	0.096
GDS-SF	0.055	0.069	0.063	0.052	0.530*	0.210	– 0.010	0.068	0.126	0.156
Diabetes	0.261***	0.056	0.034	0.042	0.063	0.163	0.227***	0.054	0.286*	0.127
Hypertension	0.514***	0.058	0.128**	0.043	0.288	0.182	0.389***	0.063	0.094	0.132
Atrial fibrillation	0.314***	0.065	0.239***	0.048	0.368*	0.176	0.093	0.063	– 0.067	0.138
Anemia	0.249***	0.061	0.145**	0.045	0.230	0.169	0.140*	0.059	– 0.383**	0.133
Hyperparathyroidism	– 0.012	0.055	0.020	0.041	0.313	0.167	0.088	0.055	0.059	0.146
CIRS-G severity index	0.297***	0.066	0.197***	0.050	0.231	0.192	0.189**	0.067	0.100	0.159
MMSE	0.005	0.010	– 0.000	0.008	0.002	0.029	0.009	0.010	– 0.017	0.023
BADL	0.034*	0.015	0.004	0.011	– 0.030	0.039	– 0.005	0.015	0.075*	0.030
IADL	0.024***	0.005	0.001	0.004	0.036**	0.013	0.014**	0.005	0.016	0.011
Constant	4.543***	0.668	6.006***	0.503	4.399*	1.801	3.871***	0.686	3.504*	1.394
lns1_1_1										
Constant	– 0.568*	0.271	– 0.807**	0.271	– 0.470	0.296	– 0.334	0.269	– 0.110	0.280
lnsig_e										
Constant	0.073***	0.015	– 0.233***	0.015	0.395***	0.033	0.010	0.016	0.282***	0.028
Observations	2204		2148		472		1974		659	

*eGFR* estimated Glomerular Filtration Rate, *CIRS-G* Cumulative Illness Rating Scale, *MMSE* Mini Mental State Examination, *GDS-SF* Geriatric Depression Scale-Short Form, *BADL* Basic Activities of Daily Living, *IADL* Instrumental Activities of Daily Living, *GLM* Generalized Linear Model

Cost variables converted in ln. *p < 0.05, **p < 0.01, ***p < 0.001

Interestingly, socioeconomic predictors had a significant impact for physician visits, whose cost was higher for higher occupational levels. Cost of care was expectedly higher for people living alone (Table 5).

## Discussion

In this large multicenter cohort study of older persons / people attending outpatient clinics for CKD in Europe, total CKD costs and cost components markedly increased with increasing CKD severity and age, and widely varied across European countries. Overall, hospitalization, medications and specialist visits were the main cost items, while the unexpectedly low impact of caregiving was likely due to difficulties in data collection, which in this area were rather lacking. As in other studies, hospitalizations were found to represent the main component of medical costs, followed by medications and physician visits [18, 36].

In multivariate analyses including also clinical and functional variables, CKD severity was not significantly associated with total costs and its weight was instead captured by comorbidities, CIRS-G and IADLs.

With global population aging, the standardized prevalence of CKD and its subsequent socioeconomic burden on healthcare systems has been increasing worldwide [26]; it is estimated that total CKD costs in Europe have reached approximately the 1.3% of total healthcare costs [42]; in participants with multimorbidity, CKD was found to be the most expensive disease [19, 43]; one of the main determinants of total cost variability was represented by country of residency. Cost of life, inflation, reimbursement practices and other features impact on the cost parameters each partner provided (supplementary material—Fig. S1). As commonly found in cost distributions, the SD is high due to the fact that some participants bore no cost (zero) in several of the resources’ categories (i.e., he/she never performed a particular visit or exam), vs very high maximum values (i.e., some individuals needed repeated costly treatments or exams). That is especially true for hospitalization.

Indeed, in this study, total CKD costs were higher in Germany and The Netherlands, followed by Austria and Spain, while Italy, Israel and Poland were characterized by lower costs. The high total cost in the Netherlands was mostly related to the high unit costs of examinations and diagnostic tests. In the German sub-sample of our cohort, an average costs per person per year were 8030€ (7848€–8212€) at CKD stage 3 and 9760€ (9266€–10,255€) at CKD stage 4. The annual cost of medications was higher in Germany than in the average of our sample (1870€ vs 1083€), however, the major cost drivers were represented by hospitalizations, contributing to more than 50% of total expenditures (while it accounts for 39.8% in our multinational sample). The highest costs in Germany were concordant with previous studies, and in line with recent debates about the actual impact of abundance of hospital beds and the number of in-hospital treatments [2, 44]. On the other hand, the potential cost/benefit aspects should always be remembered. For instance, even though medication were associated with a direct cost, an optimal treatment will likely save costs if hospitalizations are avoided.

Total costs not only depended on unit cost of resources, but also on the consumption of those resources for each participant. For example, Italy (like Germany) had high hospitalization and ED unit costs. But, since the total cost of hospitalization was low in Italy, those services were actually deployed less than elsewhere. Similarly, the unit cost of caregiving was high in Israel, but considering its reduced incidence on the total cost, the share of caregiving did not notably increase the Israeli total costs. The above evidence is confirmed by previous reports: for instance, in Finland, the weight of the two main cost drivers, hospitalization and medications use, was comparable to the results of our study [45]. In Spain, ﻿during the 2015–2019 period, cumulative CKD associated costs reached 14,728.4€ (2,535€ mean cost in 2019, irrespective of CKD stages) [46]. While the mean cost for hospitalization was roughly comparable with our estimate (1,971.7€ in Spain vs 2,039€ on average across CKD levels), other cost items were lower in the Spanish study (e.g., medications, visits etc.). Hospitalization, particularly due to heart failure and CKD, was responsible for 77.1% of costs, while total medication cost accounted for 6.6% of the total cost in Spain, verses 40% and 23% of our study, respectively. A possible explanation to the varying costs were the different intensity of resources used in some of the considered components. While the number of primary care visits was the same (around 8 for both samples), the Spanish system performed a lower number of specialized visits (0.7 per participant per year vs 4), ED accesses (0.4 vs 2), and diagnostic tests (0.7 vs 4). It should also be noted that mean age in the Spanish study is lower than in our sample (76.4 vs 80.3 years).

Total costs varied significantly according to the severity of the disease, sex (higher costs for women), and with increasing age.

The relationship between total CKD costs and eGFR, at least in descriptive statistics and sociodemographic models, was also confirmed by previous studies [26, 27]. In our cohort, estimated yearly costs were 4415.99€ ± 9718.09€ overall and 6704.185€ ± 11,095.53€ for eGFR < 30 participants, respectively. Turchetti et al. [47] reported a mean value of 8077.8€ ± 6400€ for CKD stages 4–5 (eGFR < 30) but, when deducting cost items not included in our approach (such as productivity losses and other costs) their value declined to around 6878€, which was aligned to the results of this study. However, when only hospital costs were concerned (on a large SHARP dataset), there was no evidence for differences in annual hospital care costs between CKD stages 1-3B and CKD stage 4 [48]. In spite of the high prevalence of anemia, we found that only few patients (2.2% of participants with eGFR < 60; 1.4% overall) were treated using ESAs, and mostly in the Austrian setting, which prevented us to explore the ESAs impact on costs. This finding is in keeping with the high prevalence of ESAs inertia recently observed in a population of older hospitalized patients [49]. The ESAs-related increased risk of cardiovascular mortality and thrombotic events [50, 51], together with the limitation of ESAs prescribing to nephrologists and oncologists/hematologists in some countries (e.g., the majority of regions in Italy) may help to explain our results. Given the observed impact of anemia on CKD-related costs in the present study, efforts should be undertaken to minimize ESAs inertia whenever possible.

To check if the results were equation dependent, we tested other specification for eGFR calculation, e.g., using EKFC formula. In spite of different proportions of patients when using EKFC classification (see table S11 in supplementary), its impact was negligible on total costs. The results of multilevel models are essentially the same (see Tables S12 and S13).

Interestingly, the severity of eGFR reduction loses its significance in multivariate analysis, where economic burden of the disease was instead captured by comorbidities (diabetes, hypertension, atrial fibrillation, anemia), multimorbidity, and functional status as measured by IADLs. It is worth noting that this result might have changed if the sample had included more severe CKD-participants requiring dialysis. That would have caused the cost to rise sharply and, from a cost-perspective, it is important to design an optimal treatment to avoid patients coming to this stage.

Despite the significant “country effects”, the above results were confirmed after correcting for the variability among countries in a multilevel framework.

This finding corroborates the importance of multidimensional assessment of participants with CKD, as multimorbidity and functional disability have known to produce a detrimental impact on participant’s prognosis and cost of care; the economic burden of CKD was already found to increase in participants with comorbid cardiovascular and metabolic diseases [2, 52]; management of comorbidities increased cost components associated with physician visits and medications, while functional impairment in ADLs and IADLs affected care services; unmet ADL/IADLs needs have been shown to lead higher healthcare consequences and psychosocial complications in the general older population [53], but no previous report addressed its socioeconomic impact in CKD; for this reason, our findings strengthen previous evidence in this field; as such, preservation of functional impairment and adequate management of comorbidities may thus help decreasing the overall consumption on health care resources in CKD patients.

This study had some limitations which are largely depended on the heterogeneity of costs between countries, and on the difficulty of finding robust values for unit costs. Furthermore, the approach adopted did not take all the costs borne by the society into account, such as loss of productivity of the participants affected by the disease, or their next of kin caring for them [18, 35]. However, productivity loss costs were found not be particularly relevant [45]. This is truer in the geriatric sample of participants to this study. Moreover, the availability of control groups (e.g., a group of matched elderly without CKD and a group of adults with CKD) would have allowed a more precise discrimination of contribution of CKD or age-related comorbidities to the healthcare costs.

It should also be noted that differences in costs might be related to an uninvestigated level of reporting by patients of their health and social-care burden.

## Conclusion

Both the advantages and limitations of the analysis are due to the multinational character of the study. The collection of costs from different economic contexts and health systems offered a complete and broad view of the cost structure of the disease. In lights of the variability of costs, a promising extension for future research would be to understand if the clinical outcome is different in different countries in relation to the money spent (provided that these data are available). On the other hand, cost estimates were subject to considerable variability due to the heterogeneity in unit costs coming from different countries. Overall, the multinational design of the study, the rich set of dependent variables used in the models (socioeconomics plus clinical), use of CGA allowing us to investigate the multidimensional components of CKD costs, the multilevel framework, and the comparison among alternative and novel screening methods were great additions of this study to the to the literature on cost analysis of CKD.

## Supplementary Information

Below is the link to the electronic supplementary material.

Supplementary file1 (DOCX 286 KB)

## Data Availability

Data will be available for SCOPE consortium on request from the principal investigator, Fabrizia Lattanzio, Italian National Research Center on Aging (IRCCS INRCA), Ancona, Fermo and Cosenza, Italy. f.lattanzio@inrca.it.
