# Supplementary material for: Healthcare costs in relation to kidney function among older people: the SCOPE study
Source: Eur Geriatr Med. 2024 Nov 13;16(1):135–48. doi: 10.1007/s41999-024-01086-8 (PMC11850571; doi:10.1007/s41999-024-01086-8)
Supplement: Supplementary file 1 — Supplementary file1 (DOCX 286 KB) [file 41999_2024_1086_MOESM1_ESM.docx]

**Healthcare costs in relation to kidney function among older people: the SCOPE study**

**Supplementary material**

Table S1: Consumption of resources in natural unit. Category and cost items

| **Cost item** | **Category** | **Unit** | **N** | **Missing** | **Miss. (%)** | **Mean** | **SD** | **Median** | **Min** | **Max** |
| --- | --- | --- | --- | --- | --- | --- | --- | --- | --- | --- |
| GP | Physician visits | Visits | 2141 | 320 | 13 | 5.12 | 4.81 | 4 | 1 | 70 |
| Specialist | Physician visits | Visits | 1850 | 611 | 24.83 | 3.44 | 3.05 | 2 | 1 | 40 |
| ED | Emergency Dept. | Visits | 270 | 2191 | 89.03 | 1.26 | 0.62 | 1 | 1 | 6 |
| Hospitalization | Hospitalization | Days | 301 | 2160 | 87.77 | 11.25 | 14.44 | 5 | 1 | 95 |
| Drugs | Medications | Total nr. | 2304 | 157 | 6.38 | 6.68 | 3.52 | 6 | 1 | 21 |
| Diagnostic tests | Visits and exams | Total nr. | 1310 | 1151 | 46.77 | 2.95 | 2.98 | 2 | 1 | 50 |
| Lab: hematology | Visits and exams | Nr. of tests | 954 | 1507 | 61.24 | 2.21 | 2.51 | 2 | 1 | 28 |
| Lab: biochemistry | Visits and exams | Nr. of tests | 1068 | 1393 | 56.6 | 10.63 | 13.74 | 7 | 1 | 191 |
| Lab: urinanalysis | Visits and exams | Nr. of tests | 612 | 1849 | 75.13 | 1.97 | 1.63 | 1 | 1 | 14 |
| Nurse | Care services | Visits | 70 | 2391 | 97.16 | 116.43 | 112.08 | 123 | 1 | 366 |
| Physiotherapy | Care services | Visits | 464 | 1997 | 81.15 | 18.79 | 20.1 | 10 | 1 | 150 |
| Home help | Care services | Visits | 274 | 2187 | 88.87 | 20.29 | 17.79 | 13 | 1 | 184 |
| Social transport | Care services | Total nr. | 131 | 2330 | 94.68 | 8.35 | 19.37 | 2 | 1 | 156 |
| Day care center | Care services | Days | 18 | 2443 | 99.27 | 46.5 | 59.09 | 21.5 | 1 | 180 |
| Prof. Caregiver | Caregiving | Hours | 232 | 2229 | 90.57 | 17.21 | 35.88 | 6 | 0.5 | 168 |
| Informal caregiver | Caregiving | Hours | 224 | 2237 | 90.9 | 11 | 14.32 | 7 | 0.5 | 148 |

*Note*: Phis. Visits, ED, Hospitalization, Medications, Visits and exams are direct medical costs. Care services and caregiving are direct non-medical costs. Indirect costs (productivity loss) and other societal costs are not considered

Table S2: Healthcare resources: cost items, category, domain and natural unit

| **Cost item** | **Category** | **Domain** | **Unit** |
| --- | --- | --- | --- |
| General Practitioner (GP) | Physician visits | Direct medical cost | Visits |
| Specialist | Physician visits | Direct medical cost | Visits |
| Emergency Dept. (ED) | Emergency Dept. | Direct medical cost | Visits |
| Hospitalization | Hospitalization | Direct medical cost | Days |
| Drugs | Medications | Direct medical cost | Total nr. |
| Diagnostic tests | Visits and exams | Direct medical cost | Total nr. |
| Lab: hematology | Visits and exams | Direct medical cost | Nr. of tests |
| Lab: biochemistry | Visits and exams | Direct medical cost | Nr. of tests |
| Lab: urinanalysis | Visits and exams | Direct medical cost | Nr. of tests |
| Nurse home visits | Care services | Indirect medical cost | Visits |
| Physiotherapy | Care services | Indirect medical cost | Visits |
| Home help | Care services | Indirect medical cost | Visits |
| Social transport | Care services | Indirect medical cost | Total nr. |
| Day care center | Care services | Indirect medical cost | Days |
| Professional caregiver | Caregiving | Cost of caregiving | Hours |
| Informal caregiver | Caregiving | Cost of caregiving | Hours |

Table S3: Unit cost data by medical centres and global average (values in 2019 €)

|  | **IT** | **IL** | **NL** | **AT** | **SP** | **PL** | **DE** | **mean** | **SD** | **min** | **max** |
| --- | --- | --- | --- | --- | --- | --- | --- | --- | --- | --- | --- |
| General practitioner | 22.9 | 23.7 | 9.6 | 51.6 | 46.1 | 19.0 | 32.0 | 31.4 | 15.1 | 9.6 | 51.6 |
| Specialist | 53.0 | 47.4 | 190.5 | 50.8 | 105.2 | 45.0 | 251.0 | 106.0 | 77.0 | 45.0 | 251.0 |
| Emergency room | 350.0 | 233.6 | 156.5 | 107.3 | 204.6 | 46.5 | 132.9 | 179.5 | 92.7 | 46.5 | 350.0 |
| Hospitalization | 911.8 | 273.4 | 738.6 | 901.2 | 548.2 | 410.0 | 643.2 | 621.8 | 232.0 | 273.4 | 911.8 |
| Hematology | 4.0 | 0.8 | 7.0 | 4.4 | 3.5 | 3.0 | 1.4 | 3.5 | 1.9 | 0.8 | 7.0 |
| Biochemistry | 15.1 | 2.9 | 2.6 | 3.6 | 3.1 | 4.3 | 11.0 | 5.7 | 4.7 | 2.6 | 15.1 |
| Urinanalysis | 4.1 | 0.8 | 4.6 | 2.1 | 1.9 | 2.5 | 6.6 | 3.1 | 1.9 | 0.8 | 6.6 |
| Nurse home visit | 25.0 | 16.9 | 59.1 | 35.5 | 23.6 | 12.0 | 34.1 | 28.7 | 14.6 | 12.0 | 59.1 |
| Physiotherapy | 25.0 | 37.2 | 34.0 | 102.5 | 19.5 | 6.3 | 18.0 | 32.7 | 30.0 | 6.3 | 102.5 |
| Home help | 20.1 | 20.8 | 21.6 | 17.8 | 23.6 | 3.8 | 24.1 | 19.4 | 6.8 | 3.8 | 25.7 |
| Social transport | 29.0 | 4.0 | 5.0 | 5.2 | 6.9 | 5.0 | 16.5 | 9.8 | 8.7 | 4.0 | 29.0 |
| Day care center | 38.0 | 32.6 | 116.0 | 45.0 | 24.0 | 13.8 | 268.8 | 70.3 | 86.3 | 13.8 | 268.8 |
| Professional caregiver | 6.5 | 31.3 | 19.5 | 8.1 | 6.9 | 7.0 | 3.5 | 11.2 | 9.4 | 3.5 | 31.3 |
| Informal caregiver | 6.5 | 20.8 | 19.5 | 6.0 | 6.9 | 3.9 | 3.5 | 9.3 | 6.9 | 3.5 | 20.8 |

Table S4: Unit cost data for drugs and diagnostic tests by medical centres (values in 2019 €)

| **Medications** | **IT** | **IL** | **NL** | **AT** | **SP1** | **PL** | **DE** | **mean** | **SD** | **min** | **max** |
| --- | --- | --- | --- | --- | --- | --- | --- | --- | --- | --- | --- |
| ACE inhibitors, plain (C09AA) | 0.03 | 0.18 | 0.20 | 0.29 | 0.32 | 0.15 | 0.70 | 0.27 | 0.21 | 0.03 | 0.70 |
| Angiotensin II antagonists, plain (C09CA) | 0.02 | 0.22 | 0.25 | 0.43 | 0.63 | 0.17 | 1.51 | 0.46 | 0.50 | 0.02 | 1.51 |
| Beta blocking agents, selective (C07AB) | 0.04 | 0.15 | 1.87 | 0.40 | 0.17 | 0.51 | 0.11 | 0.46 | 0.64 | 0.04 | 1.87 |
| Dihydropyridine derivatives (C08CA) | 0.04 | 0.13 | 0.45 | 0.38 | 0.47 | 0.10 | 0.36 | 0.27 | 0.18 | 0.04 | 0.47 |
| HMG CoA reductase inhibitors (C10AA) | 0.02 | 0.75 | 0.23 | 0.29 | 0.78 | 0.14 | 1.26 | 0.49 | 0.45 | 0.02 | 1.26 |
| Platelet aggregation inhibitors excl. Heparin (B01AC) | 0.06 | 1.34 | 0.05 | 0.32 | 0.83 | 0.60 | 0.05 | 0.46 | 0.49 | 0.05 | 1.34 |
| Proton pump inhibitors (A02BC) | 0.20 | 0.25 | 0.44 | 0.29 | 0.44 | 0.10 | 1.55 | 0.47 | 0.49 | 0.10 | 1.55 |
| Sulfonamides, plain (C03CA) | 0.01 | 0.12 | 0.06 | 0.60 | 0.13 | 0.08 | 1.31 | 0.33 | 0.47 | 0.01 | 1.31 |
| Thyroid hormones (H03AA) | 0.01 | 0.20 | 0.86 | 0.05 | 0.05 | 0.04 | 0.22 | 0.20 | 0.30 | 0.01 | 0.86 |
| Vitamin D and analogues (A11CC) | 9.03 | 0.13 | 0.32 | 0.48 | 7.87 | 0.69 | 8.36 | 3.84 | 4.30 | 0.13 | 9.03 |
| **Diagnostic tests** | **IT** | **IL** | **NL** | **AT** | **SP1** | **PL** | **DE** | **mean** | **SD** | **min** | **max** |
| Electrocardiogram | 18.24 | 10.51 | 47.54 | 22.05 | 18.00 | 9.26 | 20.38 | 20.86 | 12.72 | 9.26 | 47.54 |
| Routine X-ray of chest NOS | 22.62 | 18.48 | 45.11 | 26.30 | 9.00 | 10.42 | 10.49 | 20.35 | 12.79 | 9.00 | 45.11 |
| Echocardiography | 51.71 | 91.79 | 127.67 | 91.75 | 58.00 | 27.79 | 93.84 | 77.51 | 33.44 | 27.79 | 127.67 |
| Diagnostic ultrasound of full abdomen | 39.11 | 29.33 | 95.19 | 47.90 | 62.00 | 16.21 | 58.97 | 49.82 | 25.68 | 16.21 | 95.19 |
| Computerized axial tomography of head | 47.25 | 146.92 | 131.00 | 153.77 | 76.00 | 69.48 | 209.83 | 119.18 | 57.49 | 47.25 | 209.83 |
| Skeletal X-ray of pelvis and hip | 22.62 | 18.48 | 46.38 | 39.56 | 9.00 | 9.26 | 31.48 | 25.25 | 14.51 | 9.00 | 46.38 |
| Carotid Color Doppler ultrasonography | 51.71 | 29.74 | 257.19 | 54.70 | 53.00 | 37.06 | 120.65 | 86.29 | 80.94 | 29.74 | 257.19 |
| Skeletal x-ray of thigh, knee, and lower leg | 22.62 | 18.48 | 41.43 | 34.38 | 9.00 | 9.26 | 31.48 | 23.81 | 12.53 | 9.00 | 41.43 |
| Urine culture | 8.26 | 1.02 | 18.38 | 6.12 | 2.48 | 4.63 | 5.36 | 6.61 | 5.70 | 1.02 | 18.38 |
| Color Doppler ultrasonography of kidneys and adrenal glands | 51.71 | 29.74 | 166.30 | 60.56 | 58.00 | 27.79 | 91.15 | 69.32 | 47.76 | 27.79 | 166.30 |

Figure S1: Cost components by medical centres (distributional box plots)

Figure S2: eGFR mean (above) and percent of patients by eGFR levels (below) by medical centre

Table S5: Mean total cost by demographic characteristics of respondents (main categories, 6-months total, 2019 €)

|  | **Gender** | | | | **Age** | | | | | | | |
| --- | --- | --- | --- | --- | --- | --- | --- | --- | --- | --- | --- | --- |
|  | **male** |  | **female** |  | **75-79** |  | **80-84** |  | **85-89** |  | **90+** |  |
|  | ***mean*** | ***sd*** | ***mean*** | ***sd*** | ***mean*** | ***sd*** | ***mean*** | ***sd*** | ***mean*** | ***sd*** | ***mean*** | ***sd*** |
| Physician visits | 417.25 | 431.18 | 407.92 | 503.51 | 408.26 | 517.71 | 391.89 | 380.64 | 448.72 | 416.14 | 565.13 | 622.54 |
| Hospitalization | 835.48 | 4,276.95 | 983.09 | 4,072.65 | 621.85 | 3,219.93 | 1,189.60 | 5,343.81 | 1,476.85 | 4,514.53 | 1,584.22 | 4087.17 |
| Medications | 443.24 | 525.04 | 571.72 | 1,063.24 | 455.38 | 721.80 | 551.05 | 1,099.15 | 656.06 | 806.46 | 739.27 | 826.59 |
| Lab tests | 27.11 | 52.40 | 22.84 | 43.86 | 21.77 | 42.77 | 25.05 | 48.27 | 30.93 | 50.07 | 55.47 | 95.77 |
| Care services | 206.07 | 1,306.95 | 399.71 | 1862.50 | 225.00 | 1,291.35 | 236.62 | 1,058.21 | 738.57 | 2,983.81 | 1,258.92 | 3,820.40 |
| Caregiving | 13.94 | 65.33 | 36.97 | 207.49 | 12.52 | 89.08 | 28.16 | 216.30 | 70.71 | 206.80 | 121.15 | 267.66 |
| Diagnostic tests | 28.69 | 59.63 | 28.41 | 64.88 | 26.84 | 56.57 | 28.04 | 68.48 | 36.87 | 72.78 | 34.70 | 63.72 |
| **Total cost** | **1,971.77** | **4,754.87** | **2,450.66** | **5007.71** | **1,771.62** | **3,783.98** | **2,450.39** | **5,913.01** | **3,458.72** | **5,916.11** | **4,358.87** | **6,274.57** |

Table S6: Matrix of correlation among costs components and predictor variables based on CGA

|  | Total cost | Physician visits | Hospitalization | Medications | Care services |
| --- | --- | --- | --- | --- | --- |
| Gender (female) | 0.0485 |  |  | 0.0734 | 0.0586 |
| Age | 0.1320 |  | 0.0798 | 0.0888 | 0.1145 |
| Marital Status | 0.0669 |  |  |  | 0.0780 |
| Living alone |  |  | -0.0629 |  | 0.0926 |
| Education (years) | -0.1111 | -0.0482 | -0.0968 | -0.0564 |  |
| University diploma | -0.0850 | -0.0817 | -0.0625 |  | -0.0488 |
| Occupational level |  |  |  |  |  |
| Economic status |  |  |  |  |  |
| Income |  |  |  |  |  |
| eGFR | 0.0954 | 0.1124 | 0.0436 | 0.1270 | 0.0721 |
| Proteinuria | 0.0985 | 0.1153 | 0.0584 | 0.0969 | 0.0580 |
| Smoke |  |  | -0.0441 |  |  |
| GDS |  |  |  |  |  |
| Diabetes | 0.1071 | 0.0644 | 0.0627 | 0.1164 | 0.0702 |
| Hypertension | 0.0794 | 0.0488 | 0.0445 | 0.1311 |  |
| Atrial fibrillation | 0.1204 | 0.1192 | 0.0929 | 0.0796 |  |
| CIRS-G severity index | 0.1691 | 0.2508 | 0.0740 | 0.1666 | 0.1457 |
| MMSE | -0.1010 |  | -0.0623 |  | -0.1073 |
| ADL | 0.1602 |  | 0.1047 | 0.0757 | 0.1304 |
| IADL | 0.1828 | 0.0427 | 0.1288 | 0.0797 | 0.1355 |
| Drugs (current) | 0.1496 | 0.1220 | 0.0812 | 0.2554 | 0.0557 |
| Drugs (prescribed) | 0.1210 | 0.0936 | 0.0610 | 0.1516 | 0.1020 |
| Need of caregiving | 0.1652 | 0.0725 | 0.0751 | 0.1127 | 0.1843 |
| Hours of caregiving | 0.0972 |  | 0.0580 |  | 0.0430 |

*Note*: only statistically significant correlations are reported (ρ≥0.05)

Table S7: Sample characteristics: excluded patients vs final sample

|  | **Excluded patients** | **Final sample** | **p** |
| --- | --- | --- | --- |
| Female gender, n(%) | 151 (58.75) | 1229 (55.76) |  |
| Age, n(%) |  |  |  |
| 75-79 | 138 (54.12) | 1221 (55.40) |  |
| 80-84 | 70 (27.45) | 678 (30.76) |  |
| 85-89 | 35 (13.73) | 244 (11.07) |  |
| 90+ | 12 (4.71) | 61 (2.77) |  |
| Marital Status, n(%) |  |  |  |
| Single | 12 (6.59) | 120 (5.44) |  |
| Married/cohab. | 96 (52.75) | 1225 (55.58) |  |
| Divorced | 8 (4.40) | 117 (5.31) |  |
| Widowed | 66 (36.26) | 742 (33.67) |  |
| Living alone, n(%) | 10 (20.00) | 541 (24.55) |  |
| Education (years), mean | 11.32 | 11.26 |  |
| University diploma, n(%) | 41 (15.95) | 457 (20.74) |  |
| Occupational level, n(%) |  |  |  |
| Other | 20 (10.99) | 354 (16.06) |  |
| Low | 17 (9.34) | 195 (8.85) |  |
| Medium | 66 (36.26) | 871 (39.52) |  |
| High | 79 (43.41) | 784 (35.57) |  |
| Economic status, n(%) |  |  |  |
| Bad/Mediocre | 12 (6.59) | 233 (10.57) |  |
| Sufficient | 66 (36.26) | 829 (37.61) |  |
| Good/Very Good | 104 (57.14) | 1142 (51.81) |  |
| Not enough Income, n(%) | 16 (8.79) | 207 (9.39) |  |
| eGFR (BIS1), mean | 52.65 | 53.32 |  |
| Proteinuria, n(%) |  |  |  |
| <30 | 48 (81.36) | 1603 (72.73) |  |
| 30-300 | 10 (16.95) | 450 (20.42) |  |
| >300 | 1 (1.69) | 151 (6.85) |  |
| Smoke, n(%) |  |  |  |
| Former smoker | 62 (34.07) | 837 (37.98) |  |
| Current smoker | 5 (2.75) | 98 (4.45) |  |
| GDS-SF>5, n(%) | 24 (12.90) | 312 (14.16) |  |
| Diabetes, n(%) | 49 (25.39) | 556 (25.23) |  |
| Hypertension, n(%) | 150 (77.72) | 1704 (77.31) |  |
| Atrial fibrillation, n(%) | 21 (10.88) | 342 (15.52) |  |
| CIRS-G severity index, mean | 1.53 | 1.52 |  |
| MMSE, mean | 28.31 | 27.85 | 0.0232 |
| BADL, mean | 0.17 | 0.69 | 0.0475 |
| IADL, mean | 3.69 | 5.43 |  |
| Drugs (current)≥ 5, n(%) | 137 (53.73) | 1487 (67.47) | 0.000 |
| Drugs (prescribed)≥ 5, n(%) | 77 (30.20) | 1085 (49.23) | 0.000 |
| Need of caregiving, n(%) | 57 (31.32) | 411 (18.65) | 0.000 |
| Hours of caregiving, mean | 5.01 | 2.52 | 0.0148 |

*Note*: p-values obtained using Pearson’s Chi-squared test for categorical variables tests and ANOVAs for continuous variables. Two-sided p-values<0.05 were deemed statistically significant. Only significant p-values are reported

**eGFR measurement using 4-levels scale**

Table S8: Frequency and percent of patients by eGFR using 3- and 4- levels classifications

| **eGFR** | **N** | **%** | **Cum. %** | **eGFR** | **N** | **%** | **Cum. %** |
| --- | --- | --- | --- | --- | --- | --- | --- |
| 60+ | 741 | 33.62 | 33.62 | 60+ | 741 | 33.62 | 33.62 |
| 30-59 | 1,301 | 59.03 | 92.65 | 45-59 | 856 | 38.84 | 72.46 |
|  |  |  |  | 30-44 | 445 | 20.19 | 92.65 |
| <30 | 162 | 7.35 | 100.00 | <30 | 162 | 7.35 | 100.00 |
| Total | 2204 | 100 |  | Total | 2204 | 100 |  |

Table S9: Mean total cost by 4 eGFR levels (main categories, 6-months total, 2019 €)

|  | 60+ |  | 45-59 |  | 30-44 |  | <30 |  |
| --- | --- | --- | --- | --- | --- | --- | --- | --- |
|  | mean | sd | mean | sd | mean | sd | mean | sd |
| Physician visits | 344.29 | 365.18 | 401.51 | 529.06 | 504.32 | 493.50 | 524.18 | 479.42 |
| Hospitalization | 702.03 | 3373.78 | 882.99 | 4466.42 | 1178.17 | 4609.18 | 1373.36 | 4448.18 |
| Medications | 395.85 | 717.36 | 480.37 | 897.23 | 658.61 | 998.69 | 846.92 | 845.81 |
| Lab tests | 22.58 | 44.21 | 22.91 | 43.29 | 30.26 | 58.02 | 28.96 | 54.69 |
| Care services | 151.07 | 719.64 | 343.12 | 1848.42 | 458.37 | 1829.86 | 509.45 | 2633.86 |
| Caregiving | 28.10 | 231.06 | 21.27 | 99.11 | 35.80 | 139.05 | 25.07 | 68.38 |
| Diagnostic tests | 23.89 | 54.80 | 28.06 | 58.91 | 35.27 | 71.99 | 33.80 | 82.71 |
| **Total cost** | **1667.81** | **3743.14** | **2180.23** | **5257.18** | **2900.79** | **5509.14** | **3341.72** | **5476.94** |
| Observations | 741 |  | 856 |  | 445 |  | 162 |  |

Table S10: Predictors of total cost and costs items: multilevel models using 4-levels eGFR

|  | (1) |  | (2) |  | (3) |  | (4) |  | (5) |  |
| --- | --- | --- | --- | --- | --- | --- | --- | --- | --- | --- |
|  | **Total cost** |  | **Physician visits** |  | **Hospitalization** |  | **Medication** |  | **Cost of care** |  |
|  |  |  |  |  |  |  |  |  |  |  |
| eGFR (ref≥60) |  |  |  |  |  |  |  |  |  |  |
| 45-59 | -0.004 | (0.056) | 0.080 | (0.042) | -0.120 | (0.177) | 0.061 | (0.056) | -0.146 | (0.130) |
| 30-44 | 0.052 | (0.076) | 0.109 | (0.056) | -0.138 | (0.218) | 0.210^**^ | (0.075) | 0.016 | (0.166) |
| <30 | 0.014 | (0.117) | 0.137 | (0.088) | -0.559 | (0.330) | 0.343^**^ | (0.113) | -0.020 | (0.265) |
| Gender | 0.192^***^ | (0.058) | 0.003 | (0.043) | -0.140 | (0.177) | 0.261^***^ | (0.058) | -0.009 | (0.133) |
| Age | 0.013^*^ | (0.006) | -0.010^*^ | (0.005) | 0.020 | (0.018) | 0.009 | (0.006) | 0.020 | (0.014) |
| Marital Status (ref=single) |  |  |  |  |  |  |  |  |  |  |
| married | -0.013 | (0.112) | -0.008 | (0.084) | 0.191 | (0.317) | -0.013 | (0.114) | -0.084 | (0.234) |
| divorced | 0.203 | (0.141) | 0.188 | (0.105) | 0.269 | (0.400) | -0.075 | (0.141) | 0.078 | (0.278) |
| widowed | 0.049 | (0.108) | 0.063 | (0.081) | -0.033 | (0.297) | -0.091 | (0.110) | -0.011 | (0.215) |
| Living alone | 0.001 | (0.068) | -0.103^*^ | (0.051) | 0.059 | (0.214) | 0.020 | (0.067) | 0.412^*^ | (0.163) |
| Education (years) | -0.009 | (0.006) | -0.007 | (0.004) | -0.017 | (0.017) | -0.010 | (0.006) | 0.004 | (0.014) |
| Occupational level | 0.017 | (0.026) | 0.044^*^ | (0.019) | -0.095 | (0.085) | 0.034 | (0.026) | -0.030 | (0.063) |
| Economic status | -0.070 | (0.038) | -0.054 | (0.029) | 0.096 | (0.115) | -0.088^*^ | (0.039) | 0.108 | (0.083) |
| Proteinuria | 0.009 | (0.046) | 0.014 | (0.034) | 0.160 | (0.142) | 0.044 | (0.044) | 0.001 | (0.102) |
| Smoke | -0.011 | (0.043) | -0.036 | (0.032) | -0.211 | (0.137) | 0.068 | (0.043) | -0.067 | (0.096) |
| GDS | 0.056 | (0.069) | 0.064 | (0.052) | 0.529^*^ | (0.211) | -0.007 | (0.068) | 0.127 | (0.156) |
| Diabetes | 0.257^***^ | (0.056) | 0.032 | (0.042) | 0.064 | (0.164) | 0.218^***^ | (0.054) | 0.274^*^ | (0.127) |
| Hypertension | 0.511^***^ | (0.058) | 0.126^**^ | (0.043) | 0.290 | (0.183) | 0.379^***^ | (0.063) | 0.083 | (0.132) |
| Atrial fibrillation | 0.314^***^ | (0.065) | 0.239^***^ | (0.048) | 0.367^*^ | (0.176) | 0.092 | (0.062) | -0.064 | (0.138) |
| Anaemia | 0.242^***^ | (0.061) | 0.142^**^ | (0.045) | 0.231 | (0.170) | 0.122^*^ | (0.059) | -0.399^**^ | (0.134) |
| Hyperparathyroidism | -0.018 | (0.056) | 0.018 | (0.042) | 0.315 | (0.168) | 0.074 | (0.055) | 0.044 | (0.147) |
| CIRS-G severity index | 0.293^***^ | (0.066) | 0.195^***^ | (0.050) | 0.231 | (0.192) | 0.178^**^ | (0.067) | 0.085 | (0.159) |
| MMSE | 0.005 | (0.010) | -0.000 | (0.008) | 0.002 | (0.029) | 0.009 | (0.010) | -0.018 | (0.023) |
| ADL | 0.034^*^ | (0.015) | 0.004 | (0.011) | -0.030 | (0.039) | -0.005 | (0.015) | 0.074^*^ | (0.030) |
| IADL | 0.024^***^ | (0.005) | 0.001 | (0.004) | 0.036^**^ | (0.013) | 0.013^**^ | (0.005) | 0.016 | (0.011) |
| Constant | 4.611^***^ | (0.673) | 6.040^***^ | (0.507) | 4.393^*^ | (1.802) | 4.045^***^ | (0.689) | 3.739^**^ | (1.408) |
| lns1_1_1 |  |  |  |  |  |  |  |  |  |  |
| Constant | -0.571^*^ | (0.271) | -0.809^**^ | (0.271) | -0.469 | (0.296) | -0.339 | (0.269) | -0.119 | (0.281) |
| lnsig_e |  |  |  |  |  |  |  |  |  |  |
| Constant | 0.073^***^ | (0.015) | -0.233^***^ | (0.015) | 0.395^***^ | (0.033) | 0.009 | (0.016) | 0.281^***^ | (0.028) |
| Observations | 2204 |  | 2148 |  | 472 |  | 1974 |  | 659 |  |

*Note*: Cost variables converted in ln. Standard errors in parentheses. * p < 0.05, ** p < 0.01, *** p < 0.001

*Abbreviations:* estimated Glomerular Filtration Rate (eGFR); Cumulative Illness Rating Scale (CIRS-G); Mini Mental State Examination (MMSE); Geriatric Depression Scale – Short Form (GDS-SF); Basic Activities of Daily Living (BADL); Instrumental Activities of Daily Living (IADL). Generalized Linear Model (GLM)

**eGFR measurement using EKFC equation**

Table S11: Frequency and percent of patients by eGFR using BIS and EKFC formulas

| **eGFR BIS** | **N** | **%** | **Cum. %** | **eGFR EKFC** | **N** | **%** | **Cum. %** |
| --- | --- | --- | --- | --- | --- | --- | --- |
| 60+ | 741 | 33.62 | 33.62 | 60+ | 991 | 44.96 | 44.96 |
| 30-59 | 1,301 | 59.03 | 92.65 | 30-59 | 992 | 45.01 | 89.97 |
| <30 | 162 | 7.35 | 100.00 | <30 | 221 | 10.03 | 100.00 |
| Total | 2204 | 100 |  | Total | 2204 | 100 |  |

Table S12: Mean total cost by eGFR level using EKFC formula (main categories, 6-months total, 2019 €)

|  | 60+ |  | 30-59 |  | <30 |  |
| --- | --- | --- | --- | --- | --- | --- |
|  | mean | sd | mean | sd | mean | sd |
| Physician visits | 364.76 | 484.77 | 432.53 | 450.05 | 532.11 | 492.08 |
| Hospitalization | 637.54 | 3117.61 | 1092.76 | 4718.56 | 1389.09 | 5373.65 |
| Medications | 392.40 | 686.77 | 555.45 | 906.16 | 882.07 | 1240.83 |
| Lab tests | 22.24 | 41.82 | 26.15 | 50.05 | 29.54 | 61.04 |
| Care services | 206.79 | 1321.03 | 373.84 | 1699.45 | 526.58 | 2458.01 |
| Caregiving | 29.00 | 211.77 | 24.86 | 106.01 | 25.43 | 89.57 |
| Diagnostic tests | 23.71 | 52.75 | 32.66 | 66.32 | 31.66 | 81.95 |
| **Total cost** | **1676.43** | **3705.21** | **2538.26** | **5434.03** | **3416.47** | **6528.73** |
| Observations | 991 |  | 992 |  | 221 |  |

Table S13: Predictors of total cost and costs items: multilevel models using EKFC formula

|  | (1) |  | (2) |  | (3) |  | (4) |  | (5) |  |
| --- | --- | --- | --- | --- | --- | --- | --- | --- | --- | --- |
|  | **Total cost** |  | **Physician visits** |  | **Hospitalization** |  | **Medication** |  | **Cost of care** |  |
| eGFR EKFC (ref≥60) |  |  |  |  |  |  |  |  |  |  |
| 30-59 | 0.056 | (0.052) | 0.046 | (0.039) | 0.028 | (0.160) | 0.146** | (0.052) | -0.084 | (0.120) |
| <30 | 0.039 | (0.102) | 0.122 | (0.076) | -0.526 | (0.291) | 0.333*** | (0.098) | 0.029 | (0.225) |
| Gender | 0.192*** | (0.058) | 0.001 | (0.043) | -0.139 | (0.177) | 0.256*** | (0.058) | 0.000 | (0.133) |
| Age | 0.013* | (0.006) | -0.009 | (0.005) | 0.016 | (0.018) | 0.009 | (0.006) | 0.021 | (0.013) |
| Marital Status (ref=single) |  |  |  |  |  |  |  |  |  |  |
| married | -0.016 | (0.112) | -0.007 | (0.084) | 0.175 | (0.318) | -0.025 | (0.114) | -0.073 | (0.234) |
| divorced | 0.202 | (0.141) | 0.189 | (0.105) | 0.237 | (0.399) | -0.073 | (0.141) | 0.083 | (0.278) |
| widowed | 0.046 | (0.108) | 0.066 | (0.081) | -0.058 | (0.298) | -0.098 | (0.110) | -0.003 | (0.215) |
| Living alone | 0.001 | (0.068) | -0.104* | (0.051) | 0.059 | (0.214) | 0.016 | (0.067) | 0.410* | (0.164) |
| Education (years) | -0.009 | (0.006) | -0.007 | (0.004) | -0.018 | (0.017) | -0.010 | (0.006) | 0.004 | (0.014) |
| Occupational level | 0.017 | (0.026) | 0.045* | (0.020) | -0.090 | (0.085) | 0.035 | (0.026) | -0.028 | (0.063) |
| Economic status | -0.071 | (0.038) | -0.054 | (0.029) | 0.084 | (0.114) | -0.088* | (0.038) | 0.104 | (0.083) |
| Proteinuria | 0.010 | (0.046) | 0.010 | (0.034) | 0.186 | (0.143) | 0.044 | (0.044) | 0.005 | (0.103) |
| Smoke | -0.011 | (0.043) | -0.037 | (0.032) | -0.219 | (0.137) | 0.066 | (0.043) | -0.059 | (0.096) |
| GDS | 0.056 | (0.069) | 0.064 | (0.052) | 0.518* | (0.210) | -0.010 | (0.068) | 0.119 | (0.156) |
| Diabetes | 0.259*** | (0.056) | 0.036 | (0.042) | 0.047 | (0.163) | 0.224*** | (0.054) | 0.288* | (0.127) |
| Hypertension | 0.507*** | (0.058) | 0.131** | (0.043) | 0.273 | (0.181) | 0.378*** | (0.063) | 0.089 | (0.131) |
| Atrial fibrillation | 0.312*** | (0.065) | 0.243*** | (0.048) | 0.344* | (0.175) | 0.093 | (0.062) | -0.069 | (0.138) |
| Anaemia | 0.243*** | (0.061) | 0.141** | (0.045) | 0.236 | (0.170) | 0.124* | (0.059) | -0.388** | (0.134) |
| Hyperparathyroidism | -0.017 | (0.056) | 0.018 | (0.042) | 0.311 | (0.167) | 0.077 | (0.055) | 0.055 | (0.146) |
| CIRS-G severity index | 0.294*** | (0.066) | 0.198*** | (0.050) | 0.199 | (0.190) | 0.184** | (0.067) | 0.091 | (0.159) |
| MMSE | 0.005 | (0.010) | -0.000 | (0.008) | 0.002 | (0.029) | 0.009 | (0.010) | -0.017 | (0.023) |
| ADL | 0.034* | (0.015) | 0.003 | (0.011) | -0.029 | (0.039) | -0.006 | (0.015) | 0.075* | (0.030) |
| IADL | 0.024*** | (0.005) | 0.002 | (0.004) | 0.036** | (0.013) | 0.014** | (0.005) | 0.016 | (0.011) |
| Constant | 4.605*** | (0.667) | 5.971*** | (0.503) | 4.678** | (1.788) | 3.972*** | (0.684) | 3.556* | (1.399) |
| lns1_1_1 |  |  |  |  |  |  |  |  |  |  |
| Constant | -0.570* | (0.271) | -0.810** | (0.271) | -0.461 | (0.296) | -0.341 | (0.269) | -0.116 | (0.280) |
| lnsig_e |  |  |  |  |  |  |  |  |  |  |
| Constant | 0.073*** | (0.015) | -0.232*** | (0.015) | 0.394*** | (0.033) | 0.009 | (0.016) | 0.282*** | (0.028) |
| Observations | 2204 |  | 2148 |  | 472 |  | 1974 |  | 659 |  |

*Note*: Cost variables converted in ln. Standard errors in parentheses. * p < 0.05, ** p < 0.01, *** p < 0.001

*Abbreviations:* estimated Glomerular Filtration Rate (eGFR); Cumulative Illness Rating Scale (CIRS-G); Mini Mental State Examination (MMSE); Geriatric Depression Scale – Short Form (GDS-SF); Basic Activities of Daily Living (BADL); Instrumental Activities of Daily Living (IADL). Generalized Linear Model (GLM)
